# Opposing Roles of Foliar and Glandular Trichome Volatile Components in Cultivated Nightshade Interaction with a Specialist Herbivore

**DOI:** 10.1371/journal.pone.0160383

**Published:** 2016-08-24

**Authors:** Lucy Kananu Murungi, Hillary Kirwa, Daisy Salifu, Baldwyn Torto

**Affiliations:** 1 Behavioral and Chemical Ecology Unit, International Centre of Insect Physiology and Ecology, Nairobi, Kenya; 2 Department of Horticulture, Jomo Kenyatta University of Agriculture and Technology, Nairobi, Kenya; Pennsylvania State University, UNITED STATES

## Abstract

Plant chemistry is an important contributor to the interaction with herbivores. Here, we report on a previously unknown role for foliar and glandular trichome volatiles in their interaction with the specialist herbivore of solanaceous plants, the tomato red spider mite *Tetranychus evansi*. We used various bioassays and chemical analyses including coupled gas chromatography-mass spectrometry (GC/MS) and liquid chromatography coupled to quadrupole time of flight mass spectrometry (LC-QToF-MS) to investigate this interaction between cultivated African nightshades and *T*. *evansi*. We show that, whereas morphologically different cultivated African nightshade species released similar foliar volatile organic compounds (VOCs) that attracted *T*. *evansi*, VOCs released from exudates of ruptured glandular trichomes of one nightshade species influenced local defense on the leaf surface. VOCs from ruptured glandular trichomes comprising mainly saturated and unsaturated fatty acids deterred *T*. *evansi* oviposition. Of the fatty acids, the unsaturated fatty acids accounted for >40% of the oviposition deterrent activity. Our findings point to a defense strategy in a plant, based on opposing roles for volatiles released by foliar and glandular trichomes in response to attack by a specialist herbivore.

## Introduction

The evolutionary arms race between plants and herbivorous arthropods has been studied extensively in the past, demonstrating that plants use a variety of defensive systems against attacking herbivores [1–5]. In turn, experimental evidence has shown that herbivores can overcome these defenses through a variety of ways including avoidance [6] and/or suppression of the induction of the defense system especially through the use of various chemicals [7,8]. An example of a plant defense system against herbivory is trichomes, which vary in type, shape and mechanism of action. Apart from serving as a physical barrier to arthropod attack, trichome chemistry, especially that of glandular trichomes, has been the focus of many researchers. Glandular trichomes produce sticky secretions from their glandular heads to disable small herbivorous arthropod movement and feeding [9–11].

The physical nature or the chemical composition of the sticky secretion, and sometimes a combination of both, may hinder movement and also exert a deleterious effect on the herbivore. The chemistry of sticky secretions include foliar volatile and non-volatile chemicals with terpenes [12,13] and glucose esters [14,15], respectively, dominating these secretions in some plants. Among the terpenes, the monoterpenes *ρ*-cymene, *α*-terpinene, *α*-phellandrene, and the sesquiterpenes, zingiberene and curcumene have been shown to serve as repellents for the silverleaf whitefly, *Bemisia tabaci* Gennadius [16], whereas these same terpenes or related components may serve as attractants for other herbivores [17]. Methyl ketones such as 2-undecanone and non-volatile glucose esters have been found to deter settling of the potato aphid, *Macrosiphum euphorbiae* Thomas [14]. In line with these findings, some researchers have suggested that the presence of glandular trichomes in certain plants may indicate potency of internal leaf chemistry against herbivore establishment and performance [18–20]. On the other hand, some studies have shown that not all insect species are deterred by these secretions as reported for the mirid bug, *Tupiocoris notatus* Distant (Heteroptera: Miridae), which is specifically adapted to these unique secretions [21]. Recently, it has been shown that glandular trichomes can sense herbivore contact and directly induce defensive genes regulated by the jasmonic acid pathway [22].

The interactions of glandular trichomes with some natural enemies of herbivores have also been well documented, providing evidence that these plant structures may entrap small parasitoids and predators and thus interfere with their success as natural enemies of herbivorous arthropod pests [23,24]. As such, the judicious introduction of glandular trichomes in plant breeding programs to enhance herbivore resistance has been advocated [25,26].

Because glandular trichomes serve a variety of plant defense roles, we hypothesize that while foliar volatiles serve as attractants for herbivores, in an opposing role, their glandular trichome volatiles defend the plant against certain behaviors of the attacking herbivores. We tested this hypothesis in cultivated African nightshade—tomato red spider mite *Tetranychus evansi* Baker and Pritchard interaction, focusing on oviposition behavior in the tomato red spider mite. Such a defensive system would have significant ecological meaning if a well defended plant produces copious amounts of attractive foliar volatiles that lure the herbivore to its ‘dead-end’ while growing with poorly defended neighbors. We chose to test this hypothesis using three morphologically different cultivated African nightshades, *Solanum sarrachoides* Sendtner, *S*. *villosum* Miller and *S*. *scabrum* Miller, where the role played by physical and chemical defenses against a specialist herbivore have been partially studied [20,27]. African nightshades (Solanaceae) comprise closely related leafy species which are grouped together in the ‘*Solanum nigrum*’ complex [28]. Part of this group includes *S*. *sarrachoides*, *S*. *scabrum* and *S*. *villosum*, which are consumed widely in parts of eastern and southern Africa as indigenous leafy vegetables [29].

The tomato spider mite *T*. *evansi* is an invasive specialist herbivore of solanaceous plants of Neotropical origin and since its accidental introduction to Africa some 20 years ago [30] has become an economically important pest in vegetable farming systems across the continent [31]. Previously we showed that glandular trichomes of African nightshade species physically interfered with the movement and fecundity of *T*. *evansi* [27] and that steroidal glycoalkaloids in the leaf extract of one of these species *S*. *sarrachoides* had acaricidal effect on the mites [20]. In this study, we used various behavioral assays and chemical analyses and found evidence of a unique defense system in one of the cultivated nightshades against the herbivorous spider mite.

## Materials and Methods

### Plants and mites

We planted *S*. *sarrachoides* (accession number GBK 028726; Genebank of Kenya), *S*. *scabrum* (accession number SS51) and *S*. *villosum* (accession number MW13) obtained from the World Vegetable Centre, Arusha, Tanzania. Seeds were sterilized with 1% sodium hypochlorite solution, rinsed thrice in distilled water and germinated in seedling trays in a screen house as previously described [32] at the International Centre of Insect Physiology and Ecology (*icipe*), Duduville Campus, Kasarani in Nairobi, Kenya (S01°13.140'; E036°53.440'). After 28 days, the seedlings were transplanted into 29-cm diameter pots containing a mixture of red soil: manure: sand (in the ratio of 3:2:1 v/v). We used plants that were 4–6 weeks-old in all the experiments (S1 Fig). A colony of *T*. *evansi* was maintained on *S*. *scabrum* in a mass rearing room at *icipe* (temperature, 25 ± 1°C; 65 ± 5% RH; 12:12 light: dark photoperiod). The initial colony of *T*. *evansi* was started in 2001 with individuals collected from private land owned by tomato smallholder farmers at the Mwea Irrigation Scheme, Kenya (0°42'0''S; 37°22'00''E). Permission to collect the plant samples was sought from individual farmers. The colony at *icipe* is periodically infused with field collected specimens to avoid genetic drift. We used young adult females that were 2–3 days old in all the experiments.

### Behavioral response of *T*. *evansi* to foliar volatiles

#### Olfactory assays using intact plants

We tested the olfactory choice of *T*. *evansi* females to intact plants of *S*. *sarrachoides*, *S*. *scabrum* and *S*. *villosum* in a closed-system in a glass Y-tube olfactometer (stem 8.5 cm; arms 7.5 cm and 1 cm i.d.). Compressed medical air from a tank was purified and humidified (90% relative humidity) by passing it through activated charcoal and double distilled water, respectively. The air flow was split into two halves. One half was passed through a glass chamber (ARS Gainesville, FL, USA) enclosing a potted plant (test) and into one arm of the olfactometer at a flow rate of 350 mL min^-1^, while the other half was passed through an empty glass chamber (control) at the same flow rate. A vacuum line connected to a mini-pump (USDA/ARS-CMAVE, Gainesville, FL, USA) powered by a rechargeable battery (12 V) pulled air through the two arms of the Y-shaped glass tube at a rate of 175 mL min^-1^. Two 11W daylight fluorescent bulbs placed above the olfactometer arena illuminated the area evenly.

Young adult *T*. *evansi* females were individually picked from the colony 24 h prior to the bioassay, using an artist’s brush, placed in 2 mL centrifuge vials that were ventilated by poking holes with a sharp needle and brought to the bioassay room. Respective plant species to be tested were also brought to the bioassay room at the same time to acclimatize them to the room conditions. Individual *T*. *evansi* females were assayed for plant odor perception to the three African nightshade species in a room (25 ± 1°C; 65 ± 5% RH) in two different ways; a) each plant species was assayed against a control (charcoal-purified air); and b) the three plant species were assayed against each other in pairwise sets [33]. A single female was introduced at the stem inlet of the Y-glass tube (60° angle of the stem) and allowed a maximum of 10 min to make a choice to either arm.

A positive response was recorded if the mite chose the arm with an odor source while ‘no response’ was recorded if mites failed to reach the end of the short arms within the allocated time. The mite was subsequently removed from the tube and killed on wet cotton wool. After every three spider mites were tested, the Y-tube was changed and odor sources connected to the opposite arm to reduce effects of spatial influence on choice. A total of 60 mites per odor source were tested. In the beginning of the assays, we tested 20 mites with clean air in both arms of the olfactometer to compensate for asymmetry and exclude bias in the set-up.

#### Collection of foliar headspace volatiles

We collected volatiles from three intact African nightshades species using a headspace sampling system similar to that described previously [34]. Plants were brought to the laboratory 12 h prior to volatile sampling to acclimatize them to the room conditions. Volatiles were collected onto pre-cleaned (with dichloromethane) and dried (with white spot nitrogen) adsorbent traps (7.5 cm x 0.4 cm glass tubes) containing 30 mg Super Q (80/100 mesh, Analytical Research Systems, Gainesville, FL, USA). Air flow into an oven-baked polythene bag (Baco, Wrap Film Systems Ltd., UK) was provided by two Teflon tubes using a portable battery operated pump (USDA/ARS-CMAVE, Gainesville, FL, USA). One tube pushed air into the bag over the plant foliage while the other pulled the volatiles through the adsorbent trap at a flow rate of 175 mL min^-1^ for 12 h. After volatile collection, the traps were eluted with 100 μL of gas chromatography (GC) grade dichloromethane (Sigma-Aldrich, Gillingham, UK). Eluates (100 μL) from five plants of each respective plant species were combined to constitute 500 μL of sample which were eventually reduced to 50–100 μL under a gentle stream of nitrogen with the sample vials placed in a box of ice. Samples were stored at -80°C in 1.5 mL glass vials. All collections were replicated three times with five fresh plants per replicate of each African nightshade species.

#### Analysis of foliar headspace volatiles

We analyzed aliquots of the respective eluates by gas chromatography/mass spectrometry (GC/MS) on a HP 7890A series gas chromatograph (Agilent Technologies, Wilmington, USA) linked to a HP 5975C mass spectrometer (Agilent Technologies, Wilmington, USA) operated in the electron ionization mode. The instrument was equipped with a non-polar HP-5MS capillary column (30 m x 0.25 mm i.d; 0.25 μm film thickness; J & W Scientific, Folsom, CA, USA) with 5%-phenyl methyl silicone as the stationary phase. Helium was used as the carrier gas at 1.2 mL min^-1^. One microliter of each sample was injected in the splitless mode at 35°C for 5 min, increasing to 280°C at 10°C min^-1^. The injector and the detector were held isothermal at 280°C for 10.5 min. The ion source temperature was 230°C. Electron ionization mass spectra were acquired at 70 eV within a mass range of 38–550 Daltons (Da) during a scan time of 0.73 scans sec^-1^. Volatile compounds were identified using their retention times and mass fragmentation spectra against authentic standards (those available) analyzed similarly. Others were tentatively identified using matches of three mass spectral libraries Adams, Chemoecol and National Institute of Standards and Technology (NIST) (MSD Chemstation E.02.00.493, MS HP, USA).

#### Olfactory choice assays for *T*. *evansi* using synthetic foliar volatile chemicals

To determine which of the compound classes identified from the three intact African nightshade species contributed to the behavioral responses in *T*. *evansi*, we tested synthetic standards using the same Y-tube olfactometer and procedure described above using intact plants. Since the volatile profiles of the three plants were similar (see S1 table), we formulated the synthetic blends based on the amounts of compounds identified from *S*. *scabrum* as a reference profile. A blend of 9.6 ng μL^-1^ formulated in hexane from each compound class was serially diluted to yield two additional concentrations of 0.6 and 2.4 ng μL^-1^. The composition of each compound class, concentrations and the ratios of individual compounds in the class blend are shown in Table 1. In addition, similar concentrations of the individual compound classes were tested in dose response tests. Compounds were dispensed by applying 50 μL of each of the prepared doses onto 150 mg of Luna dental roll (Roeko^®^, Langenau, Germany), which were left for 10 min at 23 ± 2°C to allow the solvent to evaporate. The control consisted of 150 mg dental rolls loaded with 50 μL of hexane only.

**Table 1:** Authentic volatile compounds and their concentrations in the class blend tested in bioassays.

Compoundclass	Compound	Volume (μL) picked from a stock of 1,000 ng uL^-1^	Concentration (ng μL^-1^) in the class blend	Ratio of compounds in the class blend
Fatty acids	Octanoic acid	9.60	3.20	1
	Hexadecanoic acid	17.76	5.92	2
Green leaf volatiles	3-Methyl-2-butenal	2.40	0.80	2
	Octanal	1.32	0.44	1
	Nonanal	12.96	4.32	10
	Decanal	11.04	3.68	8
Ketones	6-Methyl-5-hepten-2-one	9.60	3.20	1
	Geranyl acetone	19.20	6.40	2
Monoterpenoids	(+)-α-Pinene	3.60	1.20	3
	(-)-β-Pinene	1.32	0.44	1
	β-Myrcene	2.52	0.84	2
	(*S*)-(-)-Limonene	18.6	6.20	14
	Linalool	2.64	0.88	2
Esters	Methyl benzoate	12.24	4.08	1
	(-)-Isobornyl acetate	16.56	5.52	1
Benzenoids	Benzaldehyde	7.02	2.34	1
	Butylated hydroxytoluene	21.81	7.27	3
Hydrocarbons	Undecane	1.56	0.52	1
	Dodecane	1.44	0.48	1
	Pentadecane	1.38	0.46	1
	Hexadecane	5.61	1.87	4
	Heptadecane	3.12	1.04	2
	Octadecane	1.53	0.51	1
	Nonadecane	1.95	0.65	1
	Eicosane	4.44	1.48	3
	Heneicosane	2.04	0.68	1
	Docosane	2.58	0.86	2
	Tricosane	1.68	0.56	1
	Tetracosane	1.44	0.48	1
Sesquiterpenes	(+)-Longifolene	5.12	1.28	1
	(*E*)-β-Caryophellene	14.00	3.5	3
	Caryophellene oxide	17.04	4.26	3

### Bioactivity of S. sarrachoides glandular trichome exudates on *T*. *evansi*

#### Collection of trichome exudates

Trichome exudates were obtained from the long and abundant glandular trichomes (type ‘T’) of *S*. *sarrachoides* [27] using a modified method [35]. The glandular head of 60 individual trichomes per leaf were probed gently with a 40 μL glass capillary tube to release the sticky exudates which were subsequently transferred separately into a vial with either: (i) 1 mL of dichloromethane (DCM) to obtain the volatile components or; (ii) 1 mL of methanol to obtain the non-volatile components. After 20 collections (~1,200 trichomes), the exudates collected in both solvents were filtered by passing them through a Pasteur pipette plugged with a thin layer of clean glass wool, and 100 mg of anhydrous sodium sulphate to remove traces of water.

#### Effect of trichome exudates on oviposition of *T*. *evansi*

We conducted oviposition assays to evaluate the effect of the DCM and methanol glandular trichome exudate extracts from *S*. *sarrachoides* on *T*. *evansi* using a previously described method [36]. The treatment arena consisted of two adjoined glass slabs (8.0 cm x 3.5 cm), with the lower one aligned with filter paper and the upper one with two holes (1.5 cm) on both ends. The experiments were conducted in the laboratory at 23 ± 2°C and 60–70% relative humidity. Mites were preconditioned in a similar manner as those used in previous bioassays (see olfactory choice assays above). The 50 μL extracts of each extraction solvent in a micropipette, were applied uniformly to individual filter paper arenas and left in a hood for 10 and 30 min to allow the DCM and methanol, respectively, to evaporate. Control arenas were loaded with 50 μL of the respective solvents used for extraction.

Ten *T*. *evansi* females were transferred to each of the treated arenas (20 mites per slab) with an artist’s brush and covered with a microscope slide to restrain the mites. The number of eggs laid in each treatment arena was counted under a dissecting microscope (Leica M125, Leica Microsystems, Cambridge, UK) 24 h post-treatment. In order to confirm whether there were any combined effects between trichomes and exudates of *S*. *sarrachoides* on spider mite oviposition, we conducted additional oviposition assays. This was done in two ways: a) removing the exudate and leaving the trichome intact; and b) loading the *S*. *sarrachoides* trichome exudate on leaf disks of *S*. *scabrum* plants (4–6 weeks old) maintained under similar conditions as described above. In the first trial, the leaves of *S*. *sarrachoides* were washed with running distilled water for 5 min in order to wash off the exudate without damaging the trichomes, which we visibly confirmed under a dissecting microscope [37] and by chemical analysis (S2 and S3 Figs). Leaves were left to dry for 30 min in a hood. Ten female *T*. *evansi* were transferred using an artist’s brush to each leaf disk and left for 24 h (temperature 23 ± 2 C; RH: 70–80%) to lay eggs. Control leaves were not washed and received a similar number of mites. Each trial was repeated four times with a total of four leaves per replicate.

In the second trial, a leaf disk (1.5 cm i.d.) was cut out from *S*. *scabrum* and subsequently, one of similar size was cut out from *S*. *sarrachoides*. The two leaf disk’s abaxial surfaces were stacked and rubbed against each other for 30 s in order to mechanically load the glandular trichome exudates of *S*. *sarrachoides* into *S*. *scabrum*. Thereafter, the leaf disk of *S*. *scabrum* was transferred with a pair of forceps to a Petri dish with moistened cotton wool and placed with the abaxial side up. Five female *T*. *evansi* were transferred using an artist’s brush to each leaf disk and left in each treatment for 24 h to lay eggs under similar conditions as the previous trials. Control leaf disks of *S*. *scabrum* were not rubbed on those of *S*. *sarrachoides*. A total of four replicates were carried out for each of the treatments.

#### GC/MS and LC-QToF-MS analysis of glandular trichome exudates

We analyzed the DCM extract by GC/MS under similar conditions described previously (see collection and analysis of foliar volatiles section above) to determine the major constituents. The methanol extract was concentrated to dryness (2 mg) in a gentle stream of nitrogen and reconstituted in 2 mL of methanol: water (1:1 v/v), vortexed for 30 s and centrifuged at 10,000 rpm for 5 min. The supernatant was transferred to an autosampler vial and analyzed by LC-QToF-MS as described previously [20]. Chromatographic separation was achieved on an ultra-performance liquid chromatography (UPLC), Waters ACQUITY I-class system (Waters Corp., Milford, MA, USA), fitted with a Waters ACQUITY UPLC BEH C_18_ column (2.1 × 50 mm, 1.7-μm particle size, Waters Corporation, Ireland) which was heated to 40°C and with an autosampler tray cooled to 5°C. Mobile phases included water (solvent A) and acetonitrile (solvent B) each containing 0.01% formic acid.

The following gradient was used: 0–0.2 min, 10% B; 0.2–3 min, 10–90% B; 3–5 min, 90% B; 5–6 min, 90–10% B; 7 min, 10% B. The flow rate was held constant at 0.4 mL min^−1^. The injection volume was 0.2 μL. The UPLC system was interfaced by electrospray ionization (ESI) to a Waters Xevo QTOF-MS (Waters Corp., Milford, MA, USA) operated in full scan MS^e^ in positive mode. Data were acquired in resolution mode over the *m*/*z* range 100–1,200 with a scan time of 1 sec using a capillary voltage of 0.5 kV, sampling cone voltage of 40 V, source temperature of 100°C and desolvation temperature of 350°C. The nitrogen desolvation flow rate was 500 L·h^−1^. For the high-energy scan function, a collision energy ramp of 25–45 eV was applied in the T-wave collision cell using ultrahigh purity argon (≥99.999%) as the collision gas. A continuous lock spray reference compound (leucine enkephalin; [M+H] ^+^ = 556.2766) was sampled at 10 sec intervals for centroid data mass correction. The mass spectrometer was calibrated across the 50 to 1,200 Daltons mass range using a 0.5 mM sodium formate solution prepared in 90:10 2-propanol/water (vol/vol). MassLynx version 4.1 SCN 712 (Waters Corp., Milford, MA, USA) was used for data acquisition and processing.

The elemental composition was generated for every analyte. Potential assignments were calculated using the monoisotopic masses with specifications of a tolerance of 10 ppm deviation and both odd- and even-electron states possible. The number and types of expected atoms was set as follows: carbons ≤ 50; hydrogens ≤ 100; oxygens ≤ 50; nitrogens ≤ 10; chlorines ≤ 10; sulfurs ≤ 10. Data acquisition and analysis by LC−QToF−MS was based on the following defined factors: mass accuracy (ppm) = 1,000,000 × (calculated mass − accurate mass)/calculated mass. The empirical formula generated was used to predict structures that were proposed based on the online database (ChemSpider, Metlin), fragmentation pattern and comparison with authentic standards analyzed similarly.

#### Bioassays of major trichome exudate components

The oviposition deterrent activity of six saturated fatty acids (decanoic, undecanoic, dodecanoic, tetradecanoic, hexadecanoic and octadecanoic acids), four unsaturated fatty acids (myristoleic, palmitoleic, linoleic and oleic acids) and 2-undecanone on *T*. *evansi* was evaluated separately following a method previously described [36] with a few modifications. A blend of 1,000 ng μL^-1^ formulated from varying concentrations of the different groups of compounds was serially diluted to yield two additional concentrations of 100 and 10 ng μL^-1^. The composition of each group, concentrations and the ratios of individual compounds in the blend are shown in Table 2. In addition, similar concentrations of the individual compound groups were tested in dose response tests. The stock solution (1,000 ng μL^-1^) was prepared in distilled water with 5% dimethyl sulfoxide (DMSO) and 15% ethanol. Distilled water (with 5% DMSO and 15% ethanol) served as the control.

**Table 2:** Concentrations of fatty acids and 2-undecanone in the blend tested in bioassays.

Chemical group	Compound	Volume (mL) to pick from a stock of 1 mg mL^-1^	Concentration (μg mL^-1^) in the blend	Ratio of compounds in the group blend
Saturated fatty acid	Decanoic acid	0.548	353.68	145
	Undecanoic acid	0.005	4.53	2
	Dodecanoic acid	0.936	548.5	225
	Tetradecanoic acid	0.061	34.2	14
	Hexadecanoic acid	0.099	56.68	23
	Octadecanoic acid	0.004	2.44	1
Unsaturated fatty acid	Myristoleic acid	0.018	175	7
	Palmitoleic acid	2.188	437.5	17
	Linoleic acid	0.001	25	1
	Oleic acid	0.181	362.5	14.5
Ketone	2-Undecanone	0.05	1.00	1

Treatments were applied by holding a leaf disk (1.5 mm) of *S*. *scabrum* with a pair of forceps and dipping it for 1 min in 10 mL of each concentration as previously described [38] and left for 1 h to dry in a hood. Leaf disks were arranged in a petri dish (4 leaf disks) stacked with moist cotton wool. Five *T*. *evansi* females (2–4 days old) were subsequently transferred to each of the treated leaf disks with an artist’s brush and left to oviposit under room conditions (23 ± 2°C; 60–70% RH). The number of eggs laid by *T*. *evansi* 24 h post treatment was counted under the dissecting microscope. A total of four replicates, each consisting of 20 mites, were carried out for each treatment.

### Chemical standards

The synthetic standards including (1R)-(+)-α-pinene, (-)-β-pinene, β-myrcene, (*S*)-(-)-limonene, linalool, octanal, nonanal, decanal, benzaldehyde, butylated hydroxytoluene, methyl benzoate, (-)-bornyl acetate, 6-methyl-5-hepten-2-one, geranyl acetone, decanoic acid, undecanoic acid, dodecanoic acid, tetradecanoic acid, hexadecanoic acid, octadecanoic acid, myristoleic acid, palmitoleic acid, linoleic acid, oleic acid, (+)-longifolene, caryophyllene oxide, (*E*)–β-caryophyllene, undecane, dodecane, pentadecane, hexadecane and quercetin were obtained from Sigma-Aldrich (>98% purity).

### Statistical analysis

From the behavioral assay data, a preference index (PI) for the choice of the odor source by *T*. *evansi* females in test or control arms was calculated according to the formula PI = [(SS-NSS)/ (SS+NSS)]*100 where SS = the number of females responding to the test odors while NSS = number of females responding to control odors [33,39]. The PI would be zero if equal number of *T*. *evansi* females were found in each arm and 100 if all *T*. *evansi* females preferred one side of the olfactometer. Within each group, count data was subjected to chi-square (χ^2^) goodness-of-fit test, testing the hypothesis that mite’s choice of the odors was in the ratio 1:1. The number of eggs laid 24 h post treatment with the *S*. *sarrachoides* components was subjected to a generalized linear model assuming Poisson distribution error and logarithmic link function to examine the effect of treatments. The oviposition deterrent index (ODI) was calculated based on the numbers of eggs on arenas treated with other components relative to those on the control according to the formula: ODI (%) = (C-T)/(C+T)*100, where C is the number of eggs on the control and T is the number of eggs on arenas treated with other components [40]. The means and their standard errors were obtained from the regression model based on the scale of the response variable (ODI). All statistical analyses were implemented in R software version 3.0.2 [41].

## Results

### Foliar volatiles and attraction in *T*. *evansi*

**Fig 1:**
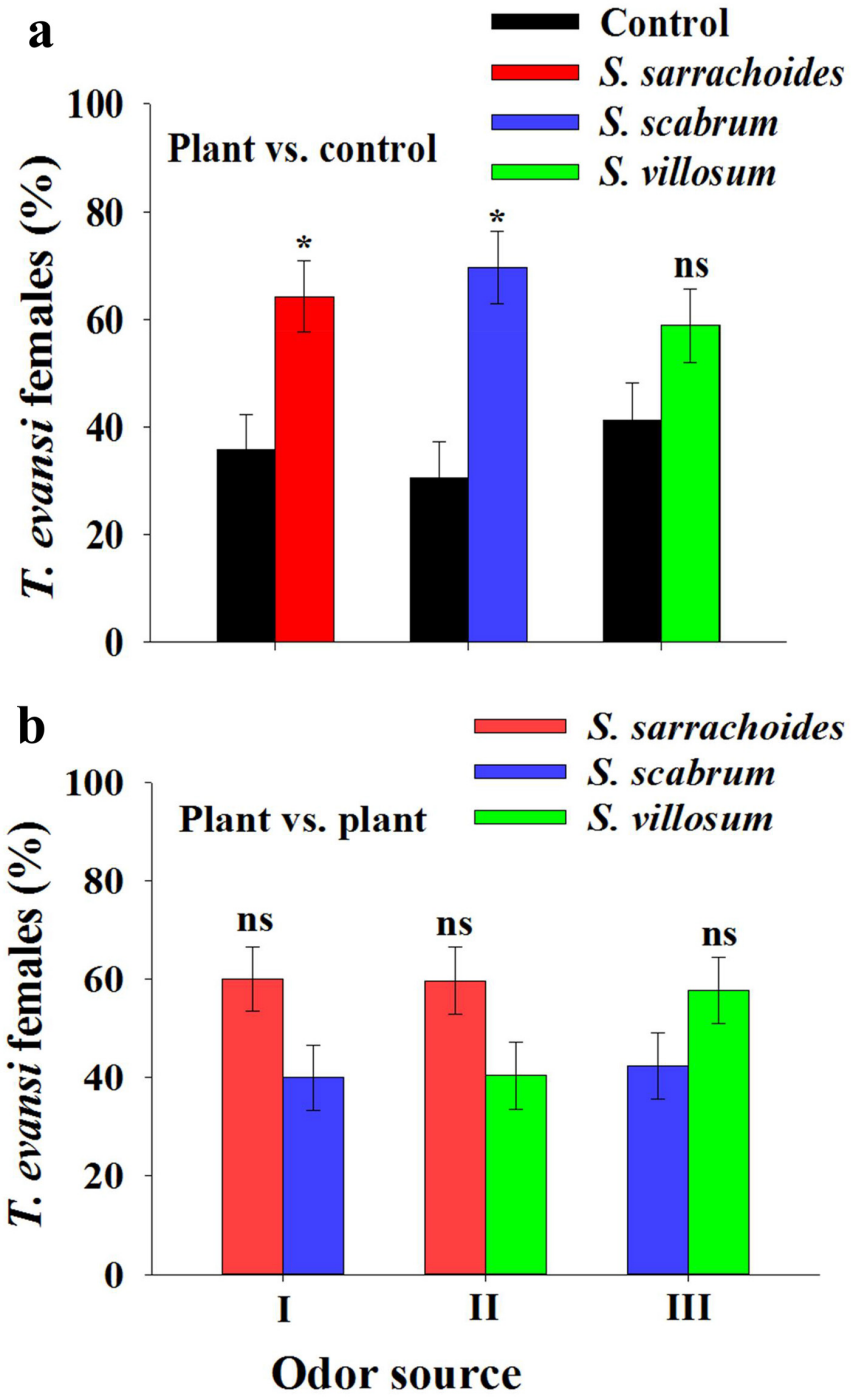
Olfactory responses of *Tetranychus evansi* females to intact plant volatile odors of three African nightshade species. (a) Intact plants compared to air (control); and b) Pairwise comparisons of intact plants. Responses are expressed as preference indices (PI); * = significant; ns = not significant (α = 0.05).

In olfactometer assays, we found that odors released by different African nightshade species attracted *T*. *evansi* but in a variable pattern (Fig 1a and 1b). When we compared plant odors with charcoal-filtered air (blank control), mites significantly preferred the odors of *S*. *sarrachoides* (χ^2^ = 3.70; P = 0.05) and *S*. *scabrum* (χ^2^ = 6.28; P = 0.01) but not those of *S*. *villosum* (χ^2^ = 1.25; P = 0.26) (Fig 1a). Despite these differences, when the plants were compared in paired assays, *T*. *evansi* did not show a significant preference for any of the odors of these African nightshade species (*S*. *sarrachoides* vs. *S*. *scabrum*: P = 0.18; *S*. *sarrachoides* vs. *S*. *villosum*: P = 0.21; *S*. *villosum* vs. *S*. *scabrum*: P = 0.33) (Fig 1b).

### Analysis of foliar volatiles

**Fig 2:**
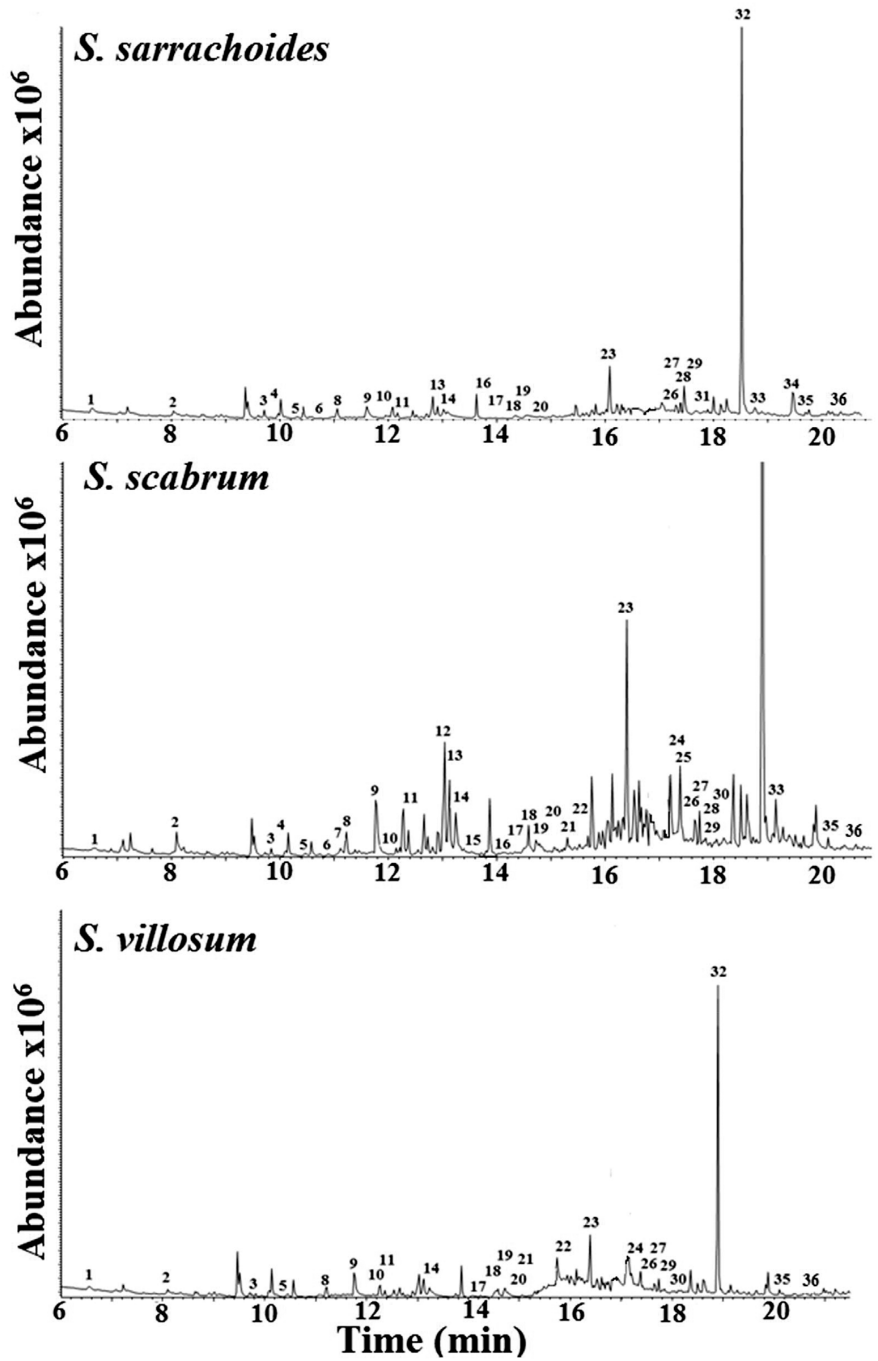
Representative chromatograms of chemical components identified in foliar volatiles of three African nightshade species *viz*. *Solanum sarrachoides*, *S*. *scabrum* and *S*. *villosum*. Peak no: 1 = hexanal; 2 = (*Z*)-3-hexen-1-ol; 3 = (+)-α-Pinene; 4 = benzaldehyde; 5 = (-)-β-Pinene; 6 = 6-methyl-5-hepten-2-one; 7 = β-myrcene; 8 = octanal; 9 = (*S*)-(-)-limonene; 10 = undecane; 11 = dihydromyrcenol; 12 = methyl benzoate; 13 = linalool; 14 = nonanal; 15 = isophorone; 16 = octanoic acid; 17 = α-terpineol; 18 = dodecane; 19 = decanal; 20 = carvacrol, methyl ether; 21 = pentadecane; 22 = bornyl acetate; 23 = hexadecane; 24 = copaene; 25 = β-elemene; 26 = longifolene; 27 = (−)-α-cedrene; 28 = (*E*)-β -caryophyllene; 29 = (+)-β-cedrene; 30 = geranyl acetone; 31 = α-humulene; 32 = butylated hydroxytoluene; 33 = δ-cadinene; 34 = caryophyllene oxide; 35 = cedrol; 36 = hexadecanoic acid.

**Fig 3:**
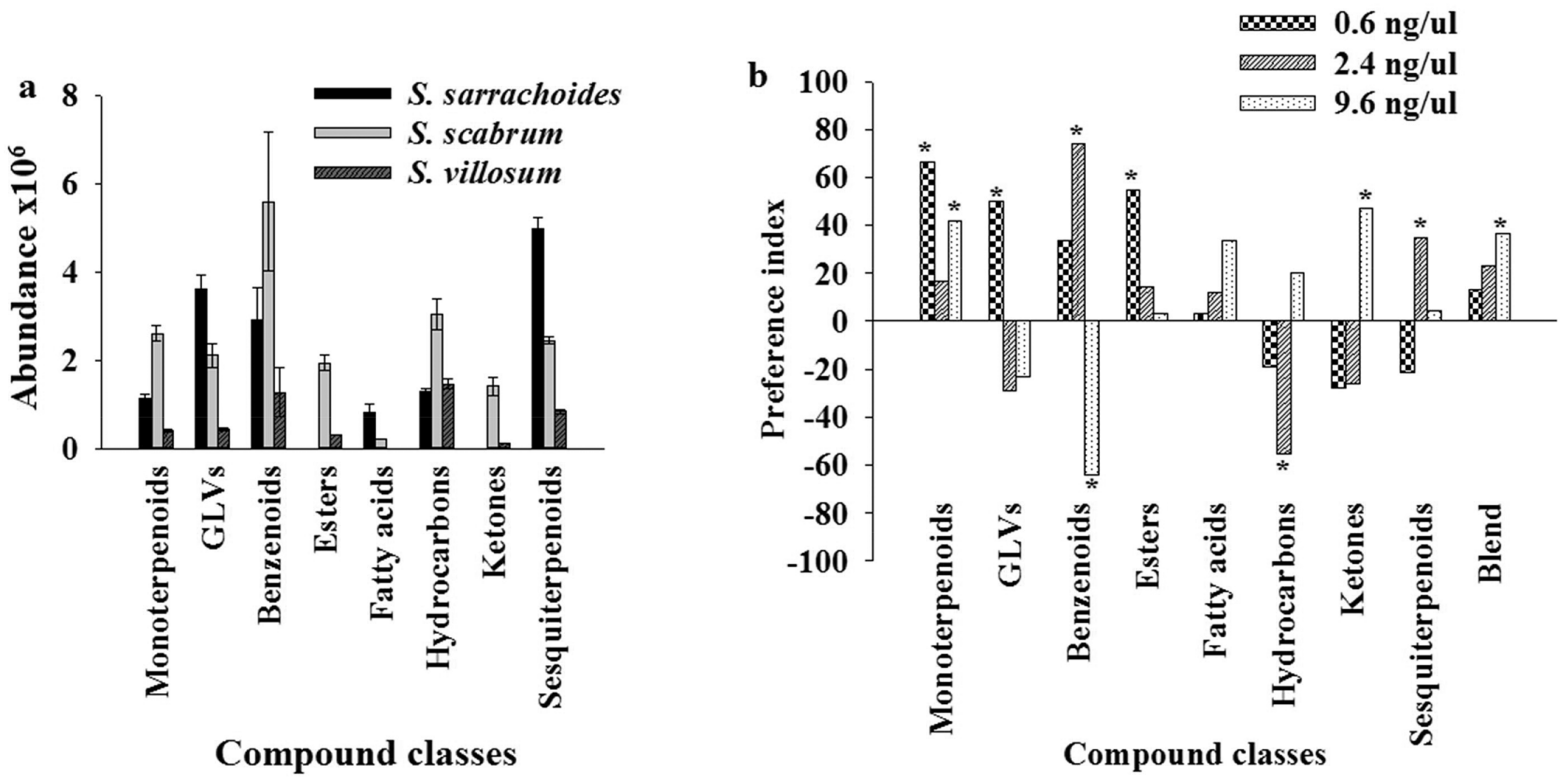
Abundance and behavioral responses of *Tetranychus evansi* to chemical components detected in intact plants of the three African nightshade species. (a) Abundance of compound classes detected from foliar scents of *Solanum sarrachoides*, *S*. *scabrum* and *S*. *villosum*; (b) olfactory response of *Tetranychus evansi* to individual compound classes and a blend of all the classes. All responses were compared to a control and expressed as preference indices; * = significant (α = 0.05).

Coupled gas chromatography/mass spectrometric analyses of volatiles released by the different African nightshade species identified similar components which varied mainly quantitatively with most components detected in *S*. *scabrum* (30) followed by *S*. *sarrachoides* (26) and the least in *S*. *villosum* (22) (Figs 2 and 3a; S1 Table). We found several chemical classes including: monoterpenoids, benzenoids, esters, hydrocarbons and ketones detected in ~2–4 fold more in *S*. *scabrum* than in *S*. *sarrachoides* and *S*. *villosum*. In addition, green leaf volatiles, fatty acids and sesquiterpenoids were twice more abundant in *S*. *sarrachoides* than they were in the volatiles of *S*. *scabrum* and *S*. *villosum* (Fig 3a; S1 Table).

### Bioassays of foliar components

In dose response assays with blends of the different classes of compounds identified in the volatiles, it was only the mite’s responses to fatty acids that followed a similar pattern of responses observed for the full blend of compounds, although not significantly different from the control. In contrast, mite response to other classes of compounds varied considerably, with some mixtures following no specific dose response pattern (Fig 3b; Table 3).

**Table 3:** χ^2^ and p-values associated with the response of adult females of *Tetranychus evansi* to synthetic blends of compound classes identified from three African nightshades species.

Compound class	Dose (ng/μL)	DF	χ^2^	P-value
Monoterpenoids	0.6	1	13.33	0.000***
	2.4	1	1.00	0.317
	9.6	1	5.45	0.020*
Green leaf volatiles (GLVs)	0.6	1	0.01	0.014*
	2.4	1	2.61	0.106
	9.6	1	2.08	0.150
Benzenoids	0.6	1	3.00	0.083
	2.4	1	17.06	0.000***
	9.6	1	11.57	0.006***
Esters	0.6	1	9.32	0.002***
	2.4	1	0.71	0.398
	9.6	1	0.03	0.858
Fatty acids	0.6	1	0.03	0.862
	2.4	1	0.47	0.493
	9.6	1	3.67	0.056
Hydrocarbons	0.6	1	1.13	0.289
	2.4	1	11.11	0.000***
	9.6	1	1.20	0.273
Ketones	0.6	1	0.10	0.096
	2.4	1	2.31	0.128
	9.6	1	7.52	0.006***
Sesquiterpenoids	0.6	1	1.48	0.223
	2.4	1	4.90	0.027*
	9.6	1	0.04	0.835
Blend	0.6	1	0.82	0.366
	2.4	1	2.82	0.093
	9.6	1	7.35	0.006

Asterisks denote a significant preference;

*** = highly significant;

* = significant (P<0.05)

### Trichomes and trichome exudates on *T*. *evansi* oviposition

**Fig 4:**
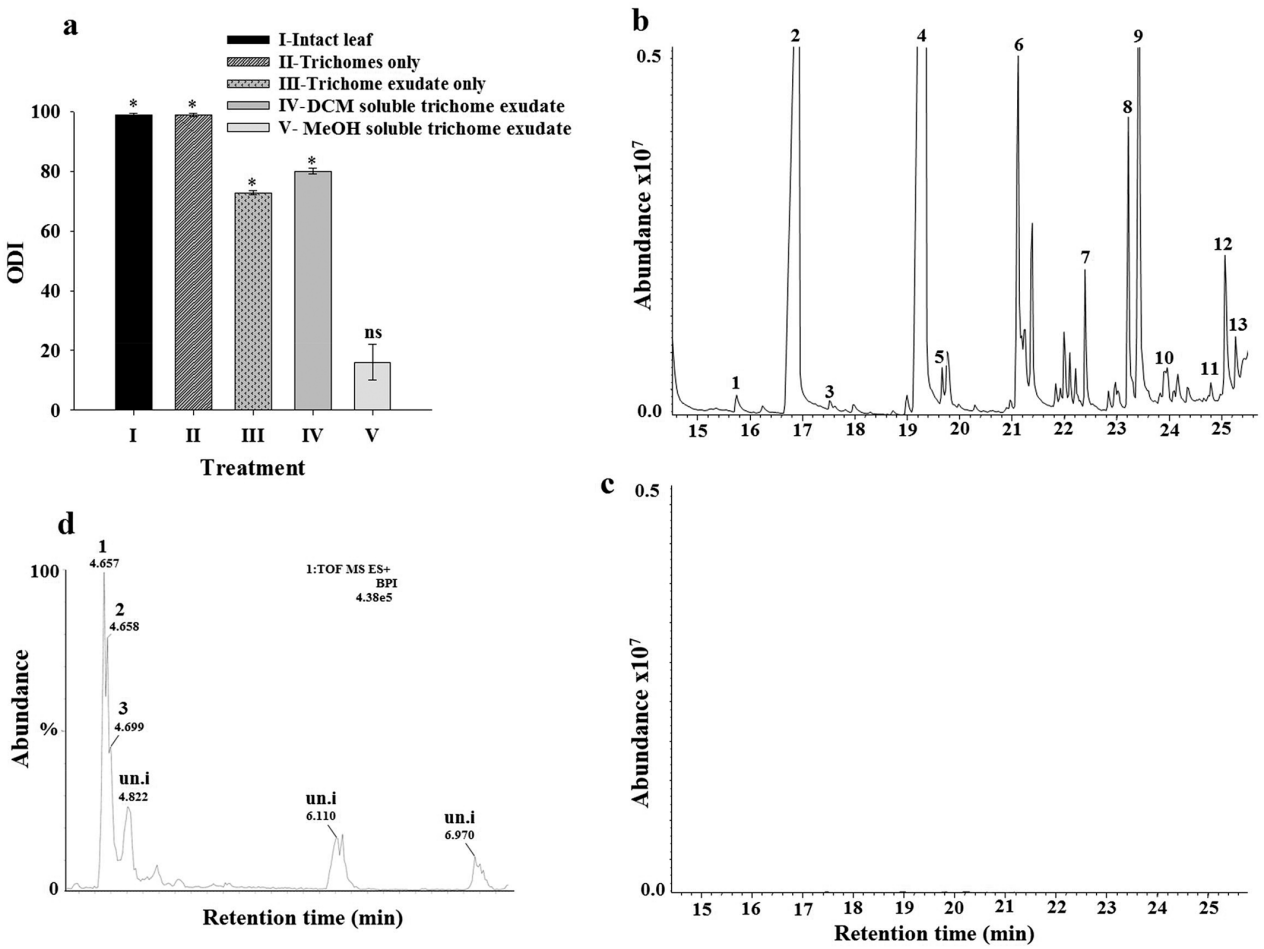
Bioactivity of glandular trichome exudates obtained from *Solanum sarrachoides* and tested on *Tetranychus evansi* females. (a) Oviposition deterrent activity of glandular trichome components of *Solanum sarrachoides* to *T*. *evansi*. Untreated leaf disks of *S*. *scabrum* were used as control in bars I, II and III. Filter paper arenas treated with dichloromethane and methanol were used as control in bars IV and V. Representative chromatograms of chemical components present in glandular trichome exudates dissolved in dichloromethane from: (b) *Solanum sarrachoides*. Peak no: 1 = 2-undecanone; 2 = decanoic acid; 3 = undecanoic acid; 4 = dodecanoic acid; 5 = myristoleic acid; 6 = tetradecanoic acid; 7 = pentadecanoic acid; 8 = palmitoleic acid; 9 = hexadecanoic acid; 10 = heptadecanoic acid; 11 = linoleic acid; 12 = oleic acid; 13 = octadecanoic acid and (c) *S*. *villosum*; and (d) representative chromatogram of chemical components present in glandular trichome exudates of *S*. *sarrachoides* dissolved in methanol; Compound no. 1 = quercetin; 2 = 6-hydroxyluteolin; 3 = hesperetin.

Since *S*. *sarrachoides* is known to harbour a high density of long glandular trichomes that reduce fecundity and movement of *T*. *evansi* [27], we tested separately whether either the trichomes or the trichome exudates deterred oviposition of *T*. *evansi*. We found that trichomes alone deterred *T*. *evansi* oviposition activity to the same level (~100%) as that elicited by the intact leaf (trichomes and exudates present) (Fig 4a). The oviposition deterrent index of the crude trichome exudate and the DCM fraction of the exudate loaded on the leaf disc of *S*. *scabrum* were similar ranging between 75% and 78% (P < 0.05). On the other hand, the oviposition deterrent index elicited by the methanol fraction of the trichome exudate was three-fold less (<20%) than that found for the DCM extract (Fig 4a).

### GC/MS and LC-QToF-MS analysis of glandular trichome exudates

GC/MS analysis identified the ketone 2-undecanone, the saturated fatty acids decanoic, undecanoic, dodecanoic, tetradecanoic, pentadecanoic, hexadecanoic, heptadecanoic and octadecanoic acids and the unsaturated fatty acids myristoleic, palmitoleic, linoleic and oleic acids in the DCM fraction of the exudate (Fig 4b). On the other hand, no detectable components were found in the DCM-soluble trichome (type ‘W’) [27] exudate extract of *S*. *villosum* (Fig 4c) analysed in a similar manner as that of *S*. *sarrachoides*. No trichome exudate data was available for *S*. *scabrum* because it lacks glandular trichomes [27]. By LC-ToF-MS analysis we detected three flavonoid aglycones in the methanol-soluble trichome exudate extract of *S*. *sarrachoides* (Fig 4d). The peaks representing compounds at retention times of 4.657, 4.658 and 4.699 min were identified as quercetin, 6-hydroxyluteolin and hesperetin, respectively (Fig 4d), with their protonated molecular ions at *m/z* 303.0507, *m/z* 303.0508 and *m/z* 303.0507, respectively (S2 Table). These assignments were based on comparison with authentic standards for quercetin and in the case of 6-hydroxyluteolin and hesperetin mass spectral data and fragmentation patterns.

### Bioassays of synthetic components identified from trichome exudates

**Fig 5:**
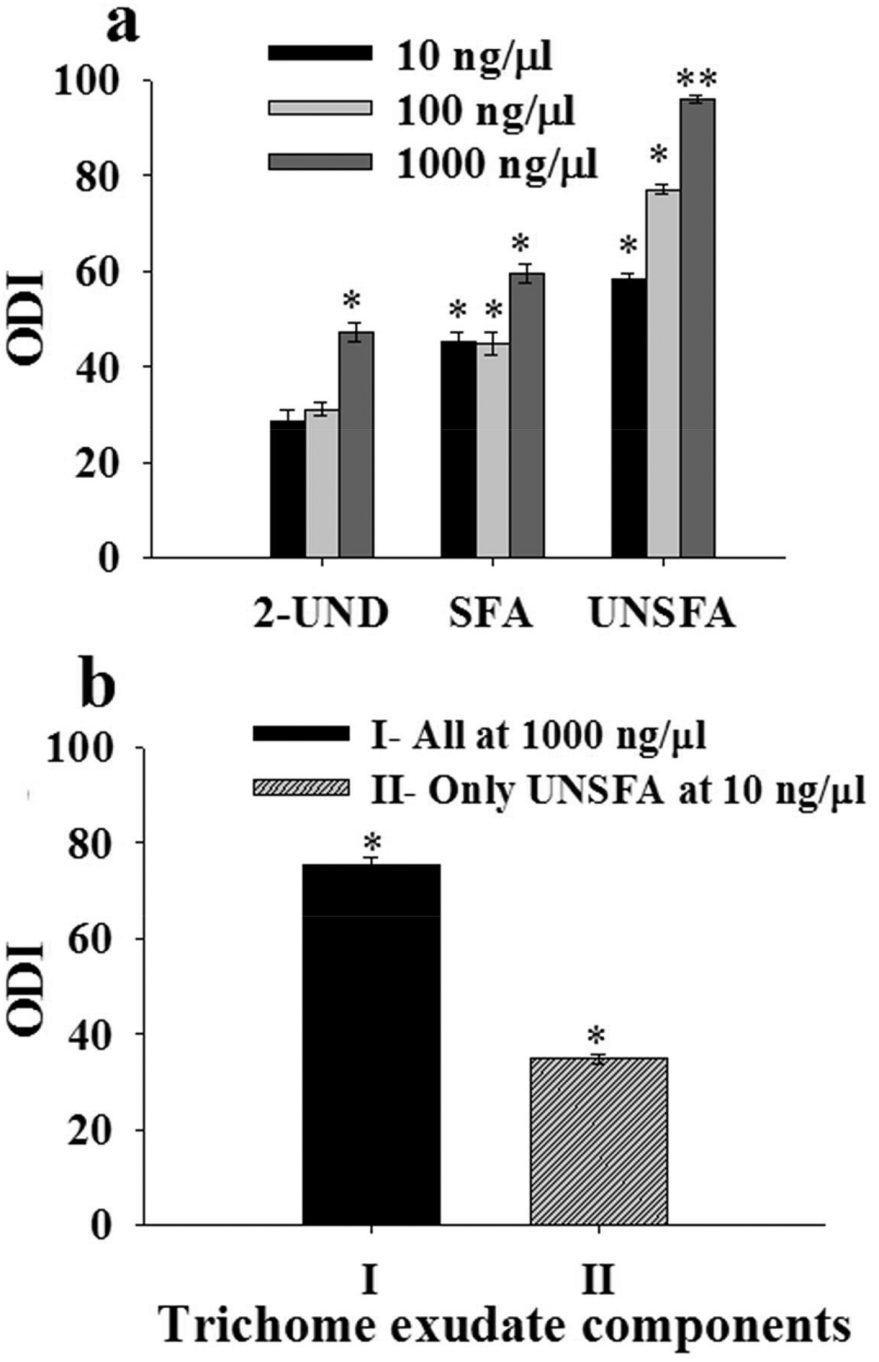
Effect of chemical components detected in trichome exudates of *S*. *sarrachoides* on oviposition deterrence activity of *Tetranychus evansi*. (a) Oviposition deterrence index (ODI) on filter paper arenas treated with blends of 2-undecanone (2-UND), saturated fatty acids (SFA) and unsaturated fatty acids (UNSFA) detected in glandular trichome exudates of *S*. *sarrachoides*. (b) Oviposition deterrence index on filter paper arenas treated with a full blend constituting; (I) 2-undecanone, saturated and unsaturated fatty acids at 1000 ng μL^-1^ and; (II) a varied concentration of the unsaturated fatty acids at 10 ng μL^-1^ whereby 2-undecanone and saturated fatty acids were maintained at 1000 ng μL^-1^.

Oviposition assays showed that the blends of saturated and unsaturated fatty acids significantly contributed to the oviposition deterrence activity observed in *S*. *sarrachoides* trichome exudate, with the latter blend being the most effective (Fig 5a). Though tested singly, 2-undecanone was less effective in deterring spider mite oviposition across all doses tested compared to the fatty acids, eliciting significant increased oviposition deterrence activity only at the highest dose of 1,000 ng μL^-1^ (Fig 5a). When we tested the full blend by reducing the concentration of the unsaturated fatty acids by 100-fold and kept the concentration of the saturated fatty acids and 2-undecanone at 1,000 ng uL^-1^, there was a >40% reduction in the oviposition deterrent activity of the blend to *T*. *evansi* (Fig 5b).

## Discussion

Previous work indicates that semiochemicals released by plants contribute to defense against invading herbivores and this may differ depending upon the plant species and the interacting herbivore [42–44]. For example, in the wild tomato *Solanum pennellii* (Correll) D'Arcy, volatile terpenoids have been implicated as contributing to the resistance of this plant species to whitefly attack [16]. Interestingly, in our study with related *Solanum* species comprising three African nightshade species, which also release terpenoids, we observed the opposite interactive effect between the resistant cultivar *S*. *sarrachoides* and the tomato red spider mite. This nightshade species produced qualitatively similar and *T*. *evansi* attractive volatiles as the mite-susceptible heterospecifics, but like the mite-susceptible species *S*. *scabrum*, it released relatively high levels of terpenoids, especially sesquiterpenoids. In bioassays, we found that *T*. *evansi* responses to the different classes of chemicals including mono- and sesqui- terpenoids, GLVs, benzenoids, hydrocarbons, ketones, short- and long-chain fatty acids varied with the chemical class, with only the fatty acids eliciting weak attractive responses from the red spider mite at all the doses tested. However, the general pattern of dose responses we found suggested that each chemical class contributed a certain degree of attraction to *T*. *evansi*. Evidently, the full blend of chemicals was the most attractive, confirming the importance of all classes of chemicals contributing to the semiochemical nature of the plant volatiles. However, it would be interesting to test combinations of the different chemical classes (binary or multiple classes) to determine whether any of these combinations would simulate the responses elicited by the full blend. Given the fact that the red spider mite responded similarly to the volatiles of the three morphologically different nightshade species, suggests that these volatiles inform it on their suitability as a host plant [45,46]. Thus it appears that foliar volatiles play little role in the chemical defense of the three morphologically different African nightshades.

As expected, morphological defense was quite efficient in deterring oviposition as demonstrated in *S*. *sarrachoides* which has glandular trichomes on its leaf surface and which deterred ~100% oviposition in the mites 24 h after they had been exposed to them. Interestingly, when we loaded the leaves of *S*. *scabrum*, which lacks trichomes, with the trichome exudates obtained from *S*. *sarrachoides*, the leaves of this species became unattractive for *T*. *evansi* egg laying by >70%, suggesting that in addition to physical defense provided by the trichomes, oviposition deterring chemical factors in this nightshade species were also present in the glandular trichomes. In a previous study, similar oviposition deterrent activity (97%) was observed in the potato tuber moth, *Phthorimaea opercullela* Zeller when the trichome contents of *Solanum berthaultii* Hawkes were transferred on to the leaflets of a susceptible potato accession [47]. Thus, our results suggest that *S*. *sarrachoides* defends itself against herbivore attack by using a combination of physical and chemical defenses, a trait that may have been inherited from an ancestral stock.

Our chemical analysis of the trichome exudates of *S*. *sarrachoides* showed the detection of low levels of the ketone 2-undecanone and relatively high levels of saturated fatty acids compared to unsaturated fatty acids. We found that a blend of the *S*. *sarrachoides* trichome exudate components formulated in the same ratio as the natural volatile extract elicited a similar oviposition deterrent activity as that found for the crude trichome exudate. However, in dose-response assays using the different chemicals classes of compounds detected in the volatile extract of the trichome exudate, the ketone 2-undecanone elicited the lowest oviposition deterrent activity; the saturated fatty acid blend was moderate in activity, with the unsaturated fatty acid blend eliciting the highest oviposition deterrent activity. Interestingly, in additional assays, we found that decreasing the level of unsaturated fatty acids in the blend by 100-fold decreased the oviposition deterrent activity by half, indicating that this class of compounds contributed significantly to oviposition deterrent activity found for the crude trichome exudate. Some of the chemicals identified in the volatile extract of the trichome exudate have been reported to deter oviposition behavior in certain arthropod herbivores. For example, 2-undecanone deters oviposition of the closely related two spotted spider mite *Tetranychus urticae* Koch [48,49]. While we had shown previously that a 3-component saturated fatty acid blend (decanoic acid, dodecanoic acid and hexadecanoic acid) identified in the essential oils of *S*. *sarrachoides* poorly deterred *T*. *evansi* oviposition [36], in the present study, we found that a 6-component blend of saturated fatty acids (decanoic acid, undecanoic acid, dodecanoic acid, tetradecanoic acid, hexadecanoic acid, octadecanoic acid), of which the 3-component blend was evidently a part of it, deterred oviposition in the spider mite. Notably, this 6-component blend contained three additional fatty acids including undecanoic acid, tetradecanoic acid and octadecanoic acid which could have contributed to the overall activity of the blend. Tetradecanoic acid and octadecanoic acid are important volatiles contributing to oviposition deterrence in the Asian Corn Borer, *Ostrinia furnacalis* Guenée [50]. In other studies, a mixture of equal ratios of the saturated fatty acid hexadecanoic acid and the unsaturated fatty acid oleic acid was found to be responsible for the natural oviposition-deterring pheromone of the cotton bollworm, *Helicoverpa armigera* Hubner [51]. Also, pubescent plants containing relatively high amounts of unsaturated fatty acids were often more protected from arthropod pests than other plants because these chemicals acted as precursors of phenolic lipids that served as sticky traps and toxicants for arthropods by decreasing their fecundity [52,53]. Such differences in the amounts of fatty acids in glandular trichome exudates and conferring protection against mite attack have been reported in the mite-resistant garden geranium, *Pelargonium* x *hortorum* Bailey compared to mite-susceptible lines [54]. Thus, from an ecological point of view, our results suggest that there may be a strong link between the levels of unsaturated fatty acids detected in the trichome exudates of *S*. *sarrachoides* and the decreased oviposition preference found in *T*. *evansi*.

When we tested the methanol fraction of the trichome exudate of *S*. *sarrachoides*, it did not deter *T*. *evansi* from ovipositing on filter paper discs. LC-Q-ToF-MS analysis of this fraction identified three flavonoids including quercetin, 6-hydroxyluteolin and hesperetin, suggesting that these flavonoid aglycones play little role in the defense of the plant against *T*. *evansi* oviposition. Evidence of quercetin found in the younger leaves of *Nicotiana attenuata* Torr. Ex Wats. as a major attractant of *T*. *notatus* has been reported [18]. On the other hand, oviposition deterrence of the swallowtail butterfly *Papilio xuthus* L., on the rutaceous plant, *Orixa japonica* Thunb. was reported to be due to the presence of a flavonoid glycoside quercetin 3-*0*-(2^G^-β-D-xylopyranosylrutinoside) [55]. The same insect was stimulated to oviposit on leaves of its host *Citrus unshiu* Marc. by the presence of the flavanones naringenin and hesperetin-7-*0*-rutinoside and the flavanol quercetin-3-*0*-rutinoside (rutin) [56,57]. Perhaps depending upon the herbivore, most host plant flavonoids and their glycosides may serve as stimulants or deterrents [58]. It would, however, be interesting to investigate whether glycoside derivatives of the identified aglycones in our study would deter or stimulate oviposition of *T*. *evansi*.

From an ecological perspective, it appears that some plants such as the cultivated African nightshade *S*. *sarrachoides* might have inherited from their ancestral stock either constitutive and/or induced trichome volatiles containing high levels of unsaturated fatty acids as more than just precursors of sticky phenolic lipids but also as direct deterrents against red spider mite oviposition. The fact that mites were attracted by foliar volatiles of a cultivated nightshade and were subsequently deterred from ovipositing by its glandular trichome volatiles points to a unique mechanism of defense. It has been suggested that in co-evolution, natural selection favors herbivores that are able to overcome plant defenses and therefore plants in turn are prompted to evolve new defense mechanisms [59]. It may well be that *S*. *sarrachoides* has inherited traits to defend itself against *T*. *evansi* by combining attractive foliar scents that lead the mite to an arsenal of defensive glandular trichome features including VOCs of the ruptured glandular exudates, and it appears that *T*. *evansi* has not yet evolved to overcome this defensive strategy in *S*. *sarrachoides*.

## Conclusion

We have shown that morphologically different cultivated African nightshade species released similar foliar volatile organic compounds (VOCs) that attracted *T*. *evansi*, but VOCs from ruptured glandular trichomes comprising mainly saturated and unsaturated fatty acids deterred *T*. *evansi* oviposition. It appears that opposing roles of foliar and glandular trichome volatile components may be an evolutionary pathway adopted by certain *Solanum* species such as cultivated African nightshades to defend themselves against herbivory by *T*. *evansi*. It would be, however, interesting to investigate the extent of this strategy in other closely related nightshade species and its application in the management of herbivorous spider mites.

## Supporting Information

S1 Fig African nightshade species. a) *Solanum sarrachoides*; b) *S*. *scabrum*; and c) *S*. *villosum*. (PDF)

S2 Fig A GC-MS chromatogram of *Solanum sarrachoides* trichome exudate extract obtained after washing the leaf with distilled water. 1 = 2-methyl propanoic acid; 2 = 2-methyl butanoic acid; 3 = undecene; 4 = decanal; 5 = 2-undecanone; 6 = decanoic acid; 7 = dodecanoic acid; 8 = tetradecanamide; 9 = hexadecanamide; 10 = (*Z*)-9-octadecenamide.
(PDF)

S3 Fig A LC-QToF-MS analysis of *Solanum sarrachoides* trichome exudate extract obtained after washing the leaf with distilled water. (PDF)

S1 Table Volatile compounds identified in the odors of intact plants of *Solanum sarrachoides*, *S*. *scabrum* and *S*. *villosum*. (PDF)

S2 Table Retention time and molecular weight (g mol^-1^) of flavonoids identified in the polar fraction of *S*. *sarrachoides* trichome exudates. (PDF)

S1 File Ms-Excel raw data for experiments conducted in the study. (XLS)
