# Supplementary figures and images for: Opposing Roles of Foliar and Glandular Trichome Volatile Components in Cultivated Nightshade Interaction with a Specialist Herbivore
Source: PLoS One. 2016 Aug 24;11(8):e0160383. doi: 10.1371/journal.pone.0160383 (PMC4996519; doi:10.1371/journal.pone.0160383)

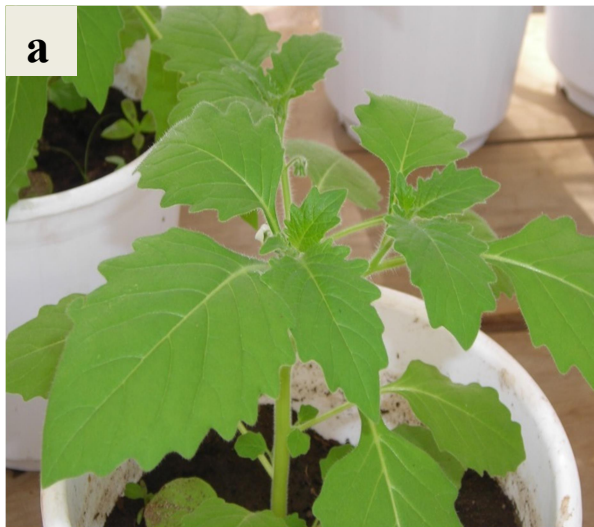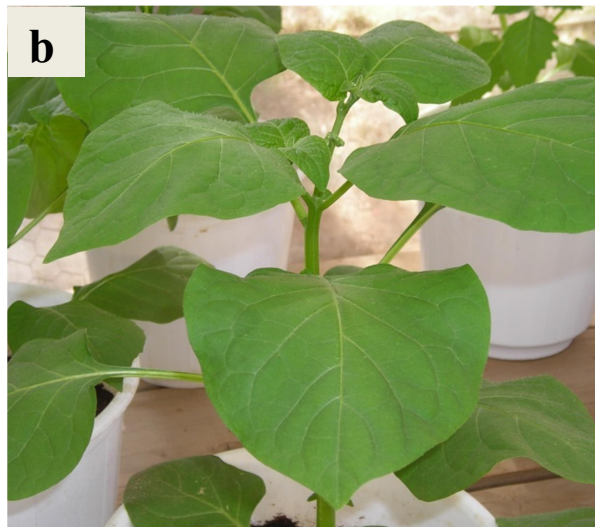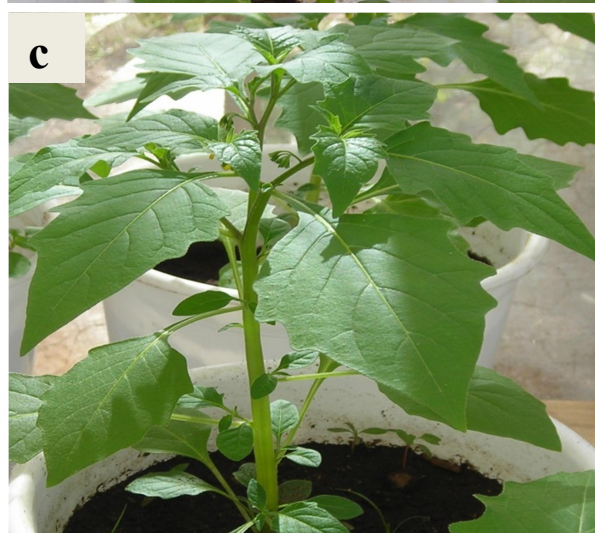

Supplement: S1 Fig — (PDF) [file pone.0160383.s001.pdf]

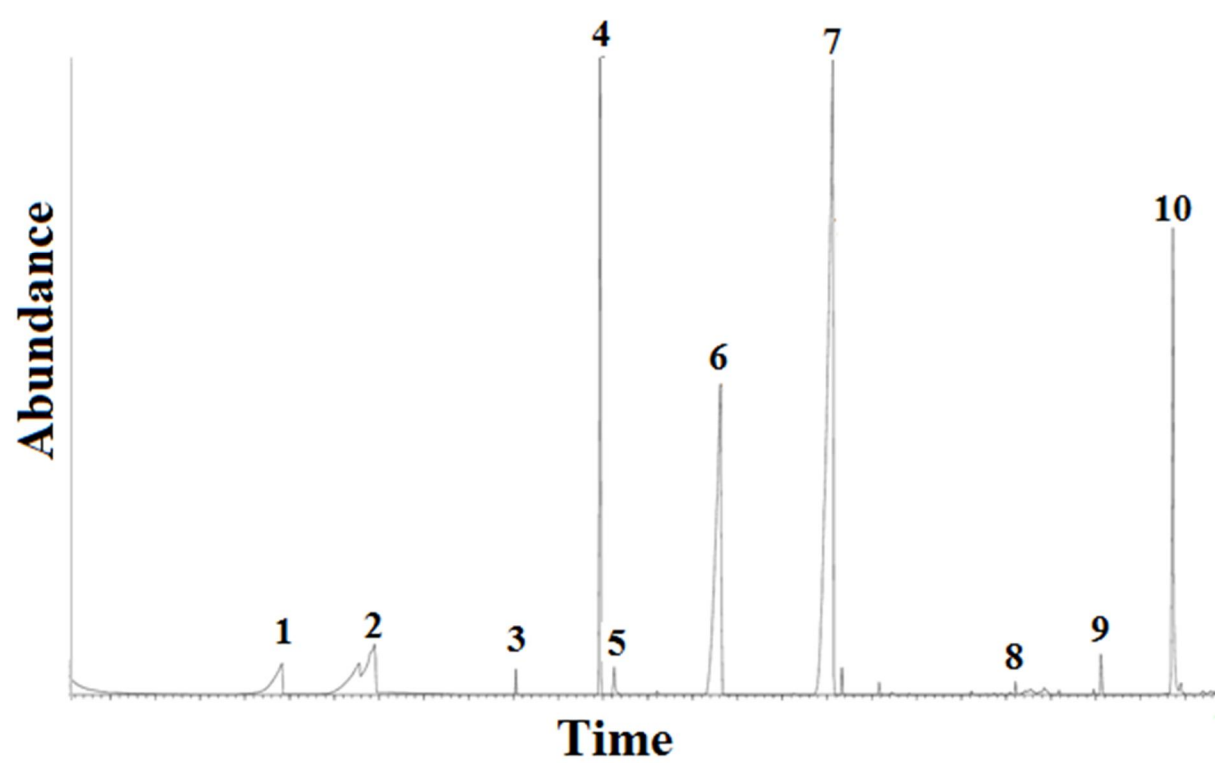

Supplement: S2 Fig — 1 = 2-methyl propanoic acid; 2 = 2-methyl butanoic acid; 3 = undecene; 4 = decanal; 5 = 2-undecanone; 6 = decanoic acid; 7 = dodecanoic acid; 8 = tetradecanamide; 9 = hexadecanamide; 10 = (Z)-9-octadecenamide. (PDF) [file pone.0160383.s002.pdf]

## Region with flavonoids

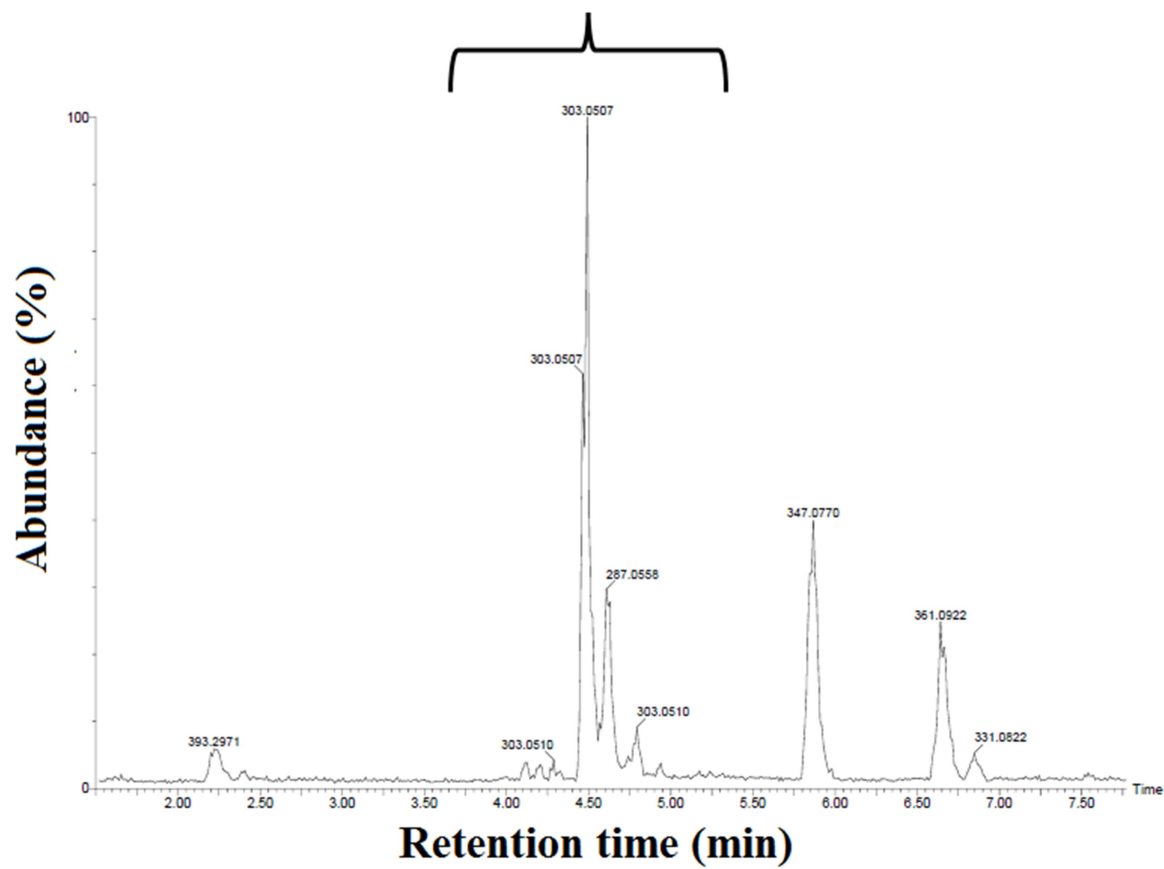

Supplement: S3 Fig — (PDF) [file pone.0160383.s003.pdf]
