# Supplementary material for: Opposing Roles of Foliar and Glandular Trichome Volatile Components in Cultivated Nightshade Interaction with a Specialist Herbivore
Source: PLoS One. 2016 Aug 24;11(8):e0160383. doi: 10.1371/journal.pone.0160383 (PMC4996519; doi:10.1371/journal.pone.0160383)
Supplement: S1 Table — (PDF) [file pone.0160383.s004.pdf]

S1 Table. Volatile compounds identified in the odors of intact plants of *Solanum sarrachoides*, *S. scabrum* and *S. villosum*

				Plant species		
**No.**	**RT**	**Compound**	**Class**	***S. sarrachoides***	***S. scabrum***	***S. villosum***
1	6.57	Hexanal	GLV	tr	tr	+
2	8.08	(*Z*)-3-hexen-1-ol	GLV	+	tr	+
3	9.80	(+)-α-Pinene*	monoterpene	+	+	+
4	10.46	Benzaldehyde*	benzenoid	+	+	-
5	10.72	(-)-β-Pinene*	monoterpene	+	+	tr
6	10.99	6-Methyl-5-hepten-2-one*	ketone	tr	+	-
7	11.08	β-Myrcene*	monoterpene	-	+	-
8	11.28	Octanal*	GLV	+	+	+
9	11.75	(*S*)-(-)-Limonene*	monoterpene	+	+	+
10	12.24	Undecane*	hydrocarbon	+	+	+
11	12.53	Dihydromyrcenol	monoterpene	+	+	+
12	13.03	Methyl benzoate*	ester	-	+	-
13	13.05	Linalool*	monoterpene	+	+	-
14	13.07	Nonanal*	GLV	+	+	+
15	13.41	Isophorone	ketone	-	+	-
16	14.26	Octanoic acid*	fatty acid	+	+	-
17	14.30	α-Terpineol*	monoterpene	+	+	+
18	14.57	Dodecane*	hydrocarbon	+	+	+
19	14.70	Decanal*	GLV	+	+	+
20	15.29	Carvacrol, methyl ether	benzenoid	+	+	+
21	15.87	Pentadecane*	hydrocarbon	-	+	+
22	15.94	Bornyl acetate*	ester	-	+	+
23	16.52	Hexadecane*	hydrocarbon	+	+	+
24	17.19	Copaene	sesquiterpene	tr	tr	tr
25	17.39	β-Elemene	sesquiterpene	-	+	-
26	17.64	Longifolene*	sesquiterpene	+	+	+
27	17.73	(−)-α-Cedrene	sesquiterpene	+	+	+
28	17.81	(*E*)-β -Caryophyllene*	sesquiterpene	+	-	-
29	17.84	(+)-β-Cedrene	sesquiterpene	+	+	+
30	18.18	Geranyl acetone*	ketone	-	+	+
31	18.27	α-Humulene*	ester	+	-	-
32	18.89	Butylated hydroxytoluene*	benzenoid	+	+	+
33	19.07	δ-Cadinene	sesquiterpene	+	+	-
34	19.88	Caryophyllene oxide*	sesquiterpene	+	-	-
35	20.10	Cedrol	sesquiterpene	+	+	+
36	20.89	Hexadecanoic acid	fatty acid	+	+	+

tr = trace <0.01%; + = detected; - = not detected ;GLV = green leaf volatile; asterisks (*) indicate those whose identities were confirmed by co-injection. RT = retention time
