# Supplementary material for: Opposing Roles of Foliar and Glandular Trichome Volatile Components in Cultivated Nightshade Interaction with a Specialist Herbivore
Source: PLoS One. 2016 Aug 24;11(8):e0160383. doi: 10.1371/journal.pone.0160383 (PMC4996519; doi:10.1371/journal.pone.0160383)
Supplement: S2 Table — (PDF) [file pone.0160383.s005.pdf]

**S2 Table.** Retention time and molecular weight (g $mol^{-1}$) of flavonoids identified in the polar fraction of *S. sarrachoides* trichome exudates

| **RT (min)** | **Compound** | **$[M+H]^+$** | **Fragment ions (*m/z*)** |
|---|---|---|---|
| 4.657[a] | Quercetin (**1**) | 303.0507 | 285.0399 |
| | | | 257.0450 |
| | | | 229.0515 |
| | | | 275.0636 |
| | | | 247.0636 |
| | | | 219.1748 |
| 4.658[b] | 6-Hydroxyluteolin (**2**) | 303.0508 | 285.0399 |
| | | | 257.0450 |
| | | | 229.0515 |
| | | | 275.0636 |
| | | | 247.0636 |
| | | | 219.1748 |
| 4.699[b] | Hesperetin (**3**) | 303.0507 | 285.0399 |
| | | | 257.0450 |
| | | | 229.0515 |
| | | | 275.0636 |
| | | | 247.0636 |
| | | | 219.1748 |

[a] Identified by comparison with authentic standard

[b] Names suggested on basis of mass spectra database and fragmentation pattern
